# Sex-specific association of epicardial adipose tissue thickness and left ventricular hypertrophy in the older adults—cross-sectional results from the population-based AugUR study

**DOI:** 10.3389/fcvm.2026.1705319

**Published:** 2026-02-18

**Authors:** Alexander D. Schober, Klaus J. Stark, Martina E. Zimmermann, Maria A. Heinrich, Lars S. Maier, Andreas Luchner, Iris M. Heid, Alexander Dietl

**Affiliations:** 1 Department of Internal Medicine II, University Hospital Regensburg, Regensburg, Germany; 2 Department of Genetic Epidemiology, University of Regensburg, Regensburg, Germany; 3 Department of Cardiology, Krankenhaus Barmherzige Brueder Regensburg, Regensburg, Germany

**Keywords:** epicardial adipose tissue, hypertrophy, left ventricular mass, older adults, population-based

## Abstract

**Aim:**

Our study aimed to evaluate epicardial adipose tissue (EAT) thickness in old adults, identify potential factors influencing it, and explore the association between EAT and left ventricular hypertrophy (LVH) in the older adult population.

**Methods:**

We analyzed cross-sectional data from the population-based AugUR study, including subjects aged ≥70 years. Cardiac geometry and EAT thickness were measured by echocardiography.

**Results:**

Among 988 participants aged 70–95 years (55.8% men), LVH was present in 368 individuals (37.2%). EAT thickness was similar between women and men (mean ± standard deviation: 4.1 ± 2.0 mm vs. 4.2 ± 1.9 mm, *p* = 0.29) but increased with age group (70–79 years: 4.0 ± 1.8 mm, >79 years 4.5 ± 2.0 mm, *p* < 0.001). Linear regression models of potential risk factors influencing EAT thickness showed associations with age [*β* = 0.038 mm per year; 95% confidence interval (CI) = 0.014–0.063, *p* < 0.05], body mass index (BMI) (*β* = 0.097 mm per kg/m^2^, 95% CI = 0.070–0.124, *p* < 0.05), and low-density lipoprotein-cholesterol (LDL, *β* = 0.008 mm per mg/dL, 95% CI = 0.004–0.012, *p* < 0.05). Given the reported causal relationship between EAT and LVH in experimental studies, multivariable regression models were performed to evaluate the association between EAT thickness and left ventricular mass index (LVMi). EAT thickness was associated with LVMi independent of sex, age, BMI, LDL, smoking, hypertension, diabetes, and chronic kidney disease (LVMi *ß* = 2.43 g/m^2^/mm, 95% CI 1.26–3.59, *p* = <0.001; LVH OR = 1.36/mm, 95% CI 1.01–1.84, *p* < 0.05). This association was strong in men but not in women.

**Conclusion:**

EAT thickness continues to increase with age beyond 69 years. Age, BMI, and LDL-cholesterol are associated with increasing EAT thickness. Our study strengthens the experimental hypothesis of a link between EAT and LVH by translational, population-based data. However, this association is more pronounced in men than in women.

## Introduction

Cardiac metabolism plays a crucial role in maintaining continuous function of the heart. Alterations in cardiac metabolism have been implicated in the pathogenesis of different cardiomyopathies, highlighting the central role of energy production and utilization (1–3).

In recent years, epicardial adipose tissue (EAT) has received increasing attention due to its potential influence on cardiac metabolic function (4, 5). EAT is located between the myocardium and the visceral layer of the pericardium, with no membrane separating it from the myocardium. This unique anatomical position allows EAT to exert direct paracrine and vasocrine effects on cardiomyocytes by releasing inflammatory cytokines, adipokines, and free fatty acids (4). These signaling molecules can affect myocardial metabolism, inflammation, and remodeling, suggesting a potential mechanistic link between EAT and structural changes in the heart (4–8).

Experimental studies in guinea pigs have provided further evidence supporting this link (9). Specifically, it has been shown that EAT can drive left ventricular hypertrophy (LVH) through the release of cytokines (9). These findings suggest that EAT not only reflects underlying metabolic and inflammatory changes but may also actively contribute to myocardial remodeling and hypertrophy. Epidemiological studies in younger populations have confirmed an association between increased EAT thickness and LVH, further supporting the hypothesis that EAT plays a role in cardiac remodeling (10–18).

However, it remains unclear whether EAT thickness continues to increase with age in older and very old individuals—the age group with the highest prevalence of LVH. Given the rising proportion of older individuals in the population and the increasing burden of cardiovascular disease in this group, understanding the relationship between EAT and LVH in older adults is of particular clinical interest.

We aimed to establish reference values for EAT thickness in older and very old individuals, identify potential risk factors influencing EAT thickness, and assess the association between EAT and LVH in this high-risk population. To address these scientific needs, we analyzed cross-sectional population-based data from the AugUR study (Age-related diseases: understanding genetic and non-genetic influences—a study at the University of Regensburg), including participants aged >69 years, living in/around the city of Regensburg, Germany (19, 20). By translating experimental findings into an epidemiological context, this study aims to improve understanding of the role of EAT in age-related cardiac remodeling.

## Methods

### Study sample

The study protocol of the AugUR study has previously been described (19, 21, 22). In brief, the AugUR study is a prospective, population-based cohort study that recruited mobile people aged 69 years or older in the city of Regensburg, Germany, and nearby counties using a random sample provided by the local registries of residence. For the first recruitment wave between 2013 and 2015 (AugUR1), 1,133 of 5,644 contacted persons provided their consent to participate (overall response rate 20.1%). All participants acompleted a 2-h study program that included a physical examination and standardized personal interviews. For participants who received echocardiography not at recruitment but during a follow-up visit, data collected during the follow-up visit were considered baseline for this analysis.

### Ethics statement

The study protocol, study procedures, and data protection strategy were approved by the Ethics Committee of the University of Regensburg, Germany (vote 12-101-0258). Written informed consent was obtained from each included individual after being informed about the study but before study inclusion. The study was conducted in accordance with the Declaration of Helsinki.

### Assessment of medical conditions, medication intake, and blood biomarkers

Trained staff collected information on medical history, cardiovascular diseases—including presence, time of onset, and interventions—current medical regimen, smoking status, and sociodemographic factors using a standardized interview. Participants were requested to take their medication packages/blisters and medication lists to the study center. Trained staff recorded all medications currently taken by each participant (21, 23).

Heart rate and blood pressure were measured three times (using an automatic device: Omron M10-IT; Omron Healthcare, Kyoto, Japan), and the average of the second and third measurements was used in these analyses.

Biomarker analyses have been previously described (23). In brief, non-fasting blood samples were drawn in a sitting position after at least 5 min of resting. Low-density lipoprotein-cholesterol (LDL) was determined on a Beckman AU 5,400 analyzer with enzymatic tests (OSR6183, OSR6187, and OSR6116; Beckman Coulter, Krefeld, Germany). Serum creatinine was determined enzymatically using a Siemens Dimension Vista 1500 (assay ECREA, Siemens Healthcare, Erlangen, Germany).

Diabetes mellitus was defined by the prescription of oral antihyperglycemic agents or insulin, in accordance with previous studies (24, 25). Heart failure was determined by self-report. Coronary artery disease (CAD) was defined as a self-reported history of myocardial infarction, coronary bypass surgery, or percutaneous coronary intervention. Body mass index (BMI) was calculated using measured height and weight with participants wearing light clothing. LDL-cholesterol was measured from fresh blood samples, and estimated glomerular filtration rate (eGFR) was calculated using serum creatinine using the CKD-EPI 2009 formula (23).

### Evaluation of cardiac geometry and epicardial adipose tissue by echocardiography

Echocardiographic assessments in the AugUR study followed two protocols. The standard program, conducted by a trained study nurse, assessed left ventricular function and morphology of all four chambers. In 1,018 participants, transthoracic echocardiography was performed by trained staff according to a standardized operating procedure using a commercially available ultrasound unit (HP Sonos 5500 with a 2–4 MHz probe; Philips, Eindhoven, The Netherlands) (21). Recorded images were *post-hoc* analyzed with Xcelera (version R3.2LI V.3.2.1.520-2011, Philips Medical Systems, Amsterdam, The Netherlands) following a standardized protocol. Left ventricular mass was calculated using M-mode in parasternal long-axis view and the Devereux formula (26). Left ventricular end-diastolic diameter, left ventricular end-systolic diameter, interventricular septum thickness, and posterior wall thickness were measured using 2D-guided M-Mode. Epicardial adipose tissue was measured in the parasternal long axis along the free RV wall at the level of the aortic valve during end systole (Supplementary Figure S1). All EAT measurements were performed *post-hoc* by a single investigator to minimize interoperator variability. For each participant, measurements were averaged over three cardiac cycles in normal heart rhythm and over 10 cardiac cycles in arrhythmia to enhance measurement reliability. Left ventricular mass was related to body surface area according to the DuBois formula (27).

Left ventricular hypertrophy was defined as left ventricular mass index >115 mg/m^2^ in men and >95 g/m^2^ in women according to the American College of Cardiology Cardiovascular Imaging Committee and European Association of Echocardiography consensus paper (28).

Left ventricular ejection fraction (LVEF) was measured using the monoplane method of discs (modified Simpson's rule) in the apical four-chamber view (28).

Cardiac cycles were defined as the interval between the onsets of transmitral inflows, measured by pulsed-wave Doppler. Heart rate was calculated as follows:

heartrate[beatsperminute]=60,000[ms]/cardiaccyclelength[ms]

Random errors were minimized by repeating all measurements three times in regular heart rhythm and 10 times in arrhythmia.

An extended echocardiographic protocol was performed in a subgroup by a cardiology-trained physician, including additional assessments of right ventricular function and heart valves. Therefore, valvular disease data are available for only 327 participants (19). High-grade valvular disease was defined as the presence of either severe aortic valve stenosis or severe mitral valve insufficiency. Valvular diseases were assessed and graded at study inclusion according to current guidelines (29, 30).

### Statistics

Continuous variables are presented as median with 25th and 75th percentiles or as mean and standard deviation. Non-continuous variables are presented as absolute numbers and percentages of non-missing values. To evaluate factors influencing EAT, univariable linear regression analysis was performed. Subsequently, multivariable linear regression analyses were performed including variables significantly associated with EAT, with additional adjustment for age and sex.

Multivariable linear regression analysis was performed to evaluate EAT as a risk factor for increased LVMi. Therefore, known risk factors for LVH were chosen as covariates (BMI, LDL-cholesterol, smoking, hypertension, diabetes, and eGFR <60 mL/min/1.72 m^2^), alongside adjustment for age and sex (31). This model was also used for both sexes separately, thereby removing sex as a covariate. The same model was used in multivariable binary regression analyses to evaluate the association between EAT and LVH, followed by separate analyses for both sexes. Regression results are presented as *ß* estimators, with corresponding 95% confidence intervals (CIs) and *p*-values. Statistical significance was defined by a two-sided *p*-value of 0.05 or less. IBM SPSS statistics version 29 was used for statistical analyses.

## Results

### Baseline characteristics of the study sample

In 1,018 participants, transthoracic echocardiographic recordings were analyzed. In 30 cases, EAT could not be measured due to insufficient image quality (feasibility 97.1%). As a consequence, the study sample for all analyses comprised 988 participants. The median age of the participants was 77 years (P25 74 years; P75 81 years; minimum 70 years, maximum 95 years), and 44.2% were women. Diabetes was the most frequent comorbidity in both men and women (Table 1). Left ventricular hypertrophy was highly prevalent, affecting 368 participants (51.2%). Severely reduced LVEF (below 30%) was observed in only four participants. Overall, the prevalence of left ventricular hypertrophy and diastolic dysfunction was quite high in this older study population, whereas systolic dysfunction was rare.

**Table 1:** Baseline characteristics of 988 participants of the AugUR study.

Parameter	Women		Men	
	mean ± SD	*n*	mean ± SD	*n*
Sociodemographic				
Age (years)	77.6 ± 4.9	437	78.3 ± 5.1	551
Comorbidities				
Uncontrolled hypertension	29 (6.7%)	436	50 (9.1%)	551
Diabetes	87 (19.9%)	437	132 (24.0%)	551
Coronary artery disease	38 (8.7%)	437	130 (23.6%)	550
Heart failure	68 (15.6%)	435	67 (12.2%)	548
Stroke	25 (5.7%)	437	65 (11.9%)	551
Smoking	114 (26.1%)	437	327 (59.3%)	551
BMI (kg/m^2^)	27.8 ± 5.0	435	28.2 ± 3.9	545
Examination results				
Heartrate (bpm)	64.5 ± 10.5	407	62.0 ± 11.1	523
Systolic blood pressure (mmHg)	130.6 ± 18.2	436	133.1 ± 18.6	551
Diastolic blood pressure (mmHg)	77.9 ± 11.1	436	76.7 ± 10.9	551
LDL-cholesterol (mg/dL)	151.9 ± 34.7	436	139.5 ± 33.6	548
eGFR_Crea_ (mL/min/1.73 m^2^)	68.6 ± 16.6	429	66.0 ± 17.2	541
Echocardiography				
EAT (mm)	4.1 ± 2.0	437	4.2 ± 1.9	551
LVMi (g/m^2^)	94.3 ± 25.2	358	117.9 ± 32.6	418
LVEF (%)	61.9 ± 6.9	424	59.3 ± 8.0	528
LVH	194 (54.2%)	358	204 (48.8%)	418
Medication				
Antiplatelet agents	141 (32.3%)	429	231 (42.1%)	524
Vitamin K antagonists	28 (6.4%)	429	51 (9.3%)	524
DOAC	19 (4.4%)	429	37 (6.7%)	524
ACE inhibitors	120 (27.5%)	429	181 (33.0%)	524
Angiotensin-II receptor blockers	112 (25.7%)	429	123 (22.4%)	524
ß-blockers	182 (41.7%)	429	223 (40.6%)	524
Mineralocorticoid receptor antagonist	16 (3.7%)	429	18 (3.3%)	524
Digitalis glycosides	10 (2.3%)	429	11 (2.0%)	524
Metformin	51 (11.7%)	429	67 (12.2%)	524
SGLT2 inhibitor	2 (0.5%)	429	1 (0.2%)	524
Statins	126 (28.9%)	429	209 (38.1%)	524

Continuous parameters are presented as mean ± SD, and non-continuous parameters are presented as absolute numbers (% non-missing); the number of paticipiants with known parameter (*n*). SD, standard deviation; BMI, body mass index; LDL, low-density lipoprotein; eGFR, estimated glomerular filtration rate based on creatinine clearance; EAT, epicardial adipose tissue; LMVi, left ventricular mass index; LVEF, left ventricular ejection fraction; LVH, left ventricular hypertrophy; DOACs, direct oral anticoagulants; ACE, angiotensin converting enzyme.

**Figure 1:**
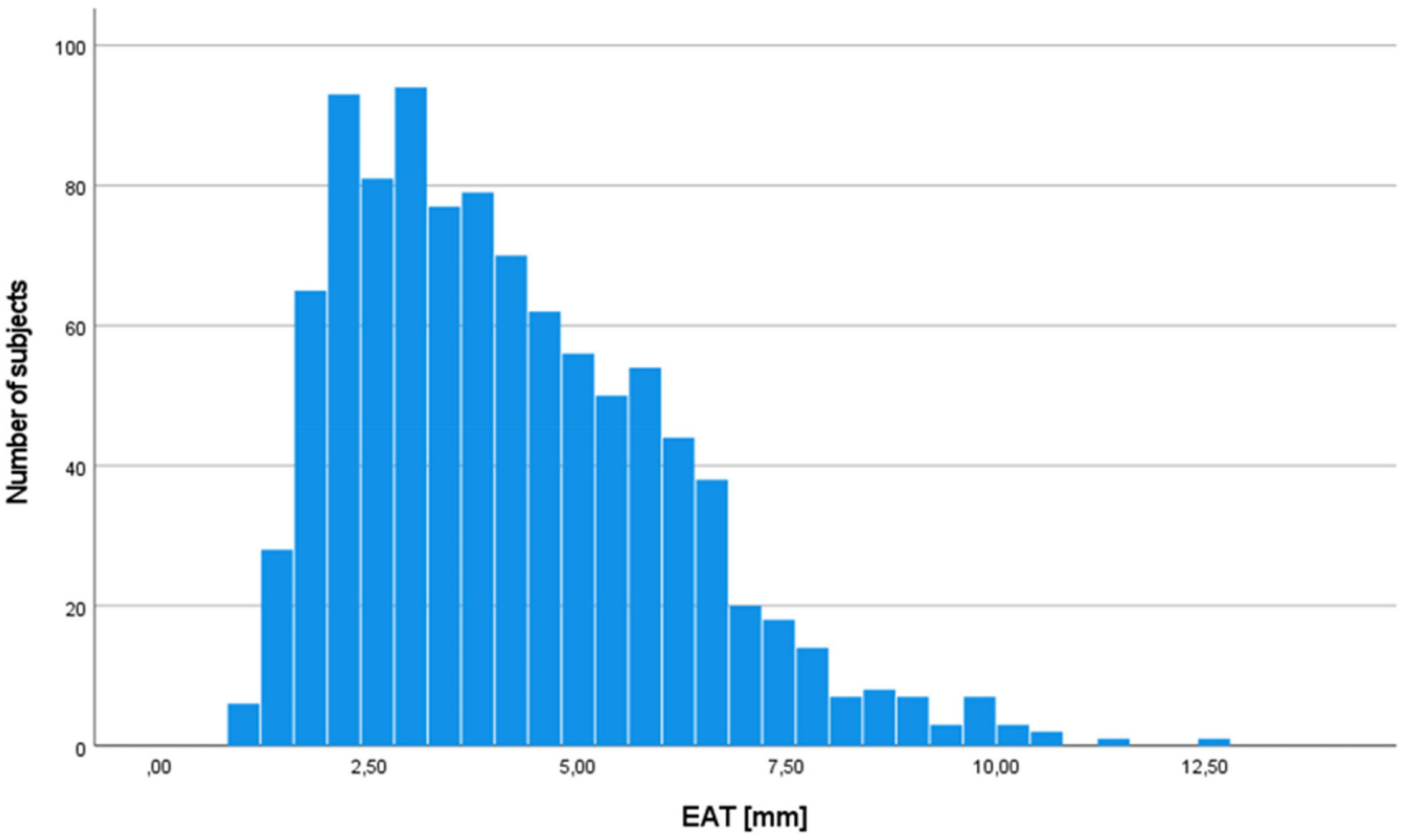
Distribution of epicardial adipose tissue in the study population (*n* = 988). EAT, epicardial adipose tissue.

EAT thickness was 4.1 ± 1.9 mm in the entire study sample, showing a mostly even distribution (Figure 1). EAT was similar in both women (4.1 ± 2.0 mm) and men (4.2 ± 1.9 mm, *p* for difference 0.29) but differed between age groups (4.0 ± 1.8 mm 70–79years, 4.5 ± 2.0 mm >80 years, *p* < 0.001).

### Factors influencing epicardial adipose tissue

To assess factors influencing EAT thickness , we evaluated established cardiovascular risk factors, which have been associated with EAT in previous trials including younger participants (hypertension, BMI, LDL-cholesterol, C-reactive protein (CRP)). In addition, the intake of metformin or statins was included, as these agents have been reported to affect EAT thickness (32–35). SGLT-2 inhibitors were not considered because only three participants were using these agents at the time of recruitment. LDL-cholesterol was included as the primary lipid parameter owing on its well-established role in cardiovascular risk and its relevance to EAT (36–38). In univariable linear regression analyses, BMI (*ß* = 0.102 mm/kg/m^2^, 95% CI 0.076–0.128, *p* < 0.001), LDL-cholesterol (*ß* = 0.004 mm/mg/dL, 95% CI 0.000–0.007, *p* = 0.045), and eGFR <60 mL/min/1.73 m^2^ (*ß* = 0.388 mm, 95% CI 0.131–0.644, *p* = 0.003) were associated with higher EAT thickness, in contrast to hypertension (*ß* = 0.126 mm/mmHg, 95% CI −0.143–0.395, *p* = 0.357), history of coronary artery disease (*ß* = 0.148 mm, 95% CI −0.168–0.465, *p* = 0.358), hs-CRP (*ß* = −0.097 mm/mg/dL, 95% CI −0.173–0.157, *p* = 0.923), or the presence of severe aortic valve stenosis (*ß* = 0.188 mm, 95% CI −2.185–2.561, *p* = 0.876) (Supplementary Table S1). Intake of metformin or statins was also associated with increased EAT thickness (metformin *ß* = 0.476 mm, 95% CI 0.11–0.842, *p* = 0.01; statin *ß* = 0.279, 95% CI 0.028–0.53, *p* = 0.03). These factors were included in a multivariable regression analysis (Table 2). BMI and LDL level remained associated with EAT thickness, independent of each other and of age and statin intake. Together, BMI and LDL level, but not hypertension, sex, ejection fraction (EF), or chronic kidney disease, are the main determinants of EAT thickness in participants aged 70 years and older, which may point to EAT thickness as a metabolic marker.

**Table 2:** Factors influencing epicardial adipose tissue.

Parameter	*ß*	95% CI	*p*-Value
Total study population (*n* = 969)			
Age (years)	0.038 mm/year	0.014–0.063	0.002
Sex (O/1 f/m)	0.086 mm	−0.151–0.323	0.477
BMI (kg/m^2^)	0.097 mm/kg/m^2^	0.070–0.124	<0.001
LDL (mg/dL)	0.008 mm/mg/dl	0.004–0.012	<0.001
eGFR <60 (O/1 n/y)	0.168 mm	−0.097–0.432	0.213
Diabetes (O/1 n/y)	0.149 mm	−0.234–0.531	0.446
Metformin (O/1 n/y)	0.090 mm	−0.385–0.566	0.710
Statin (O/1 n/y)	0.353 mm	0.080–0.626	0.011
Women (*n* = 429)			
Age (ears)	0.035 mm/year	−0.003–0.073	0.072
BMI (kg/m^2^)	0.082 mm/kg/m^2^	0.045–0.119	<0.001
LDL (mg/dL)	0.010 mm/mg/dl	0.005–0.016	<0.001
eGFR <60 (O/1 n/y)	0.234 mm	−0.177–0.644	0.265
Diabetes (O/1 n/y)	0.497 mm	−0.120–1.114	0.114
Metformin (O/1 n/y)	−0.018 mm	−0.772–0.736	0.962
Statin (O/1 n/y)	0.462 mm	0.025–0.900	0.038
Men (*n* = 540)			
Age (Years)	0.042	0.010–0.074	0.011
BMI (kg/m^2^)	0.113	0.073–0.154	<0.001
LDL (mg/dL)	0.006	0.001–0.011	0.023
eGFR <60 (O/1 n/y)	0.139	−0.209–0.487	0.432
Diabetes (O/1 n/y)	−0.102	−0.595–0.392	0.685
Metformin (O/1 n/y)	0.189	−0.430–0.808	0.548
Statin (O/1 n/y)	0.251	−0.101–0.602	0.161

BMI and LDL-cholesterol are independently associated with EAT thickness in multivariable linear regression analyses. EAT, epicardial adipose tissue; BMI, body mass index; LDL, low-density lipoprotein; eGFR, estimated glomerular filtration rate; 95% CI, 95% confidence interval of ß.

### Association between epicardial adipose tissue and left ventricular mass and hypertrophy

As the paracrine activity of EAT has been shown to promote increases in LVMi and therefore LVH in animal models (9) and EAT has also been associated with left ventricular hypertrophy in younger, healthy populations (13, 15), we sought to determine whether EAT remains associated with LVMi and LVH in an older age group in whom LVH and resulting diastolic dysfunction represent common challenges in daily clinical practice. Consequently, we performed multivariable linear regression analyses to evaluate the association between EAT and LVMi. We selected established risk factors for left ventricular hypertrophy—and therefore increased LVMi—together with age and sex as covariates. In detail, the resulting multivariable regression model included EAT, sex, and age; LDL-level and BMI, both of which were associated with EAT thickness in our previous model; and coronary artery disease, hypertension, smoking, diabetes, and chronic kidney disease (eGFR <60 mL/min/1.73 m^2^), all of which are known risk factors for LVH (31, 39). EAT thickness was independently associated with left ventricular mass (*ß* = 2.43 g/m^2^/mm, 95% CI 1.26–3.59, *p* < 0.001, Table 3). Since sex was strongly associated with LVMi, we also performed regression analyses for men and women separately. Of note, EAT was independently associated with left ventricular mass in 409 men with a reasonable effect size (*ß* = 3.95 g/m^2^/mm, 95% CI 2.17–5.73, *p* < 0.001), but not at all in 350 women (*ß* = 0.79 g/m^2^/mm, 95% CI −0.67–2.25, *p* = 0.29). Overall, EAT thickness is strongly associated with LVMi in older participants, among whom left ventricular hypertrophy (51.2%) is considerably prevalent. However, the association is strong among men but not among women. These effects persisted after adjusting for median systolic and diastolic blood pressure instead of hypertension, respectively.

**Table 3:** Multivariable linear regression showing association between EAT and left ventricular mass index.

Parameter	*ß*	95% CI	*p*-Value
Total study population (*n* = 759)			
EAT	2.356	1.200–3.511	<0.001
Sex	21.496	17.108–25.883	<0.001
Age	0.601	0.167–1.035	<0.05
CAD	11.073	5.214–16.932	<0.001
BMI	1.185	0.685–1.684	<0.001
LDL	−0.007	−0.071–0.058	0.833
Smoking	−0.576	−4.905–3.754	0.794
Hypertension	9.459	2.346–16.573	<0.05
Diabetes	0.191	−5.065–5.446	0.943
eGFR <60	−3.231	−7.887–1.425	0.173
Women (*n* = 350)			
EAT	0.813	−0.647–2.274	0.274
Age	0.823	0.253–1.393	<0.05
CAD	9.206	−0.552–18.964	0.064
BMI	0.711	0.130–1.292	<0.05
LDL	−0.064	−0.144–0.016	0.115
Smoking	−0.216	−6.238–5.806	0.944
Hypertension	4.066	−6.087–14.219	0.431
Diabetes	0.809	−6.238–7.856	0.821
eGFR <60	−2.771	−8.861–3.318	0.371
Men (*n* = 409)			
EAT	3.706	1.953–5.459	<0.001
Age	0.501	−0.138–1.141	0.124
CAD	12.476	4.839–20.113	<0.05
BMI	1.786	0.947–2.624	<0.001
LDL	0.054	−0.046–0.154	0.287
Smoking	−1.249	−7.404–4.907	0.690
Hypertension	11.781	1.804–21.758	<0.05
Diabetes	−0.454	−8.126–7.217	0.907
eGFR <60	−3.404	−10.246–3.438	0.329

In linear regression, EAT is independently associated with left ventricular mass. This effect is more pronounced in male than in female participants. CI, confidence interval of ß; EAT, epicardial adipose tissue; BMI, body mass index; LDL, low-density lipoprotein; CAD, coronary artery disease; eGFR, estimated glomerular filtration rate.

**Figure 2:**
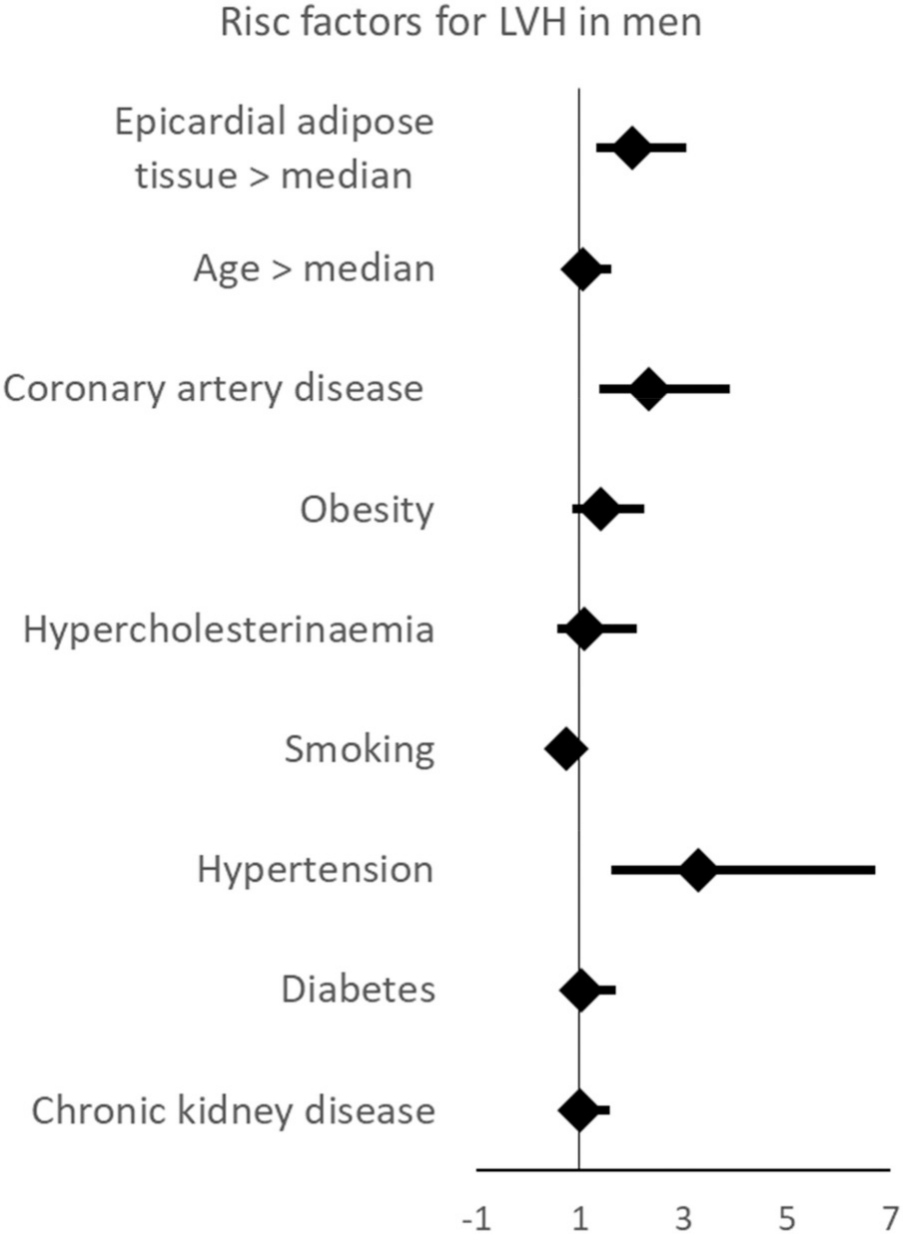
Multivariable linear regression showing that EAT is associated with left ventricular hypertrophy, and linear regression showing that EAT is independently associated with left hypertrophy. This effect is more pronounced in male than in female participants. LVH, left ventricular hypertrophy.

Next, we examined whether EAT was not only associated with LVMi but also with pathologic LVH. We performed multivariable binary logistic regression analyses using the same set of variables. Consistent with our findings for LVMi, epicardial adipose tissue was associated with LVH (Table 4). The association between EAT per mm and LVH was more pronounced than that observed between diabetes or chronic kidney disease and LVH. Thus, EAT emerged a stronger risk marker for LVH than some usually suspected comorbidities contributing to LVH. Only hypertension was more strongly associated with LVH than EAT (Figure 2). This effect was well evident in men but not in women.

**Table 4:** Multivariable binary logistic regression showing association between EAT and left ventricular hypertrophy.

Parameter	OR	95% CI	*p*-Value
Total study population (*n* = 759)			
EAT > median	1.452	1.076–1.959	<0.05
Sex	1.058	0.779–1.438	0.717
Age > median	1.102	0.801–1.515	0.551
CAD	1.579	1.125–2.217	<0.05
Obesity	1.040	0.606–1.786	0.886
Hypercholesterinemia	0.783	0.570–1.075	0.131
Smoking	2.780	1.616–4.779	<0.001
Hypertension	1.274	0.872–1.862	0.210
Diabetes	1.042	0.745–1.457	0.811
eGFR <60	2.100	1.363–3.234	<0.001
Women (*n* = 350)			
EAT > median	0.993	0.638–1.547	0.977
Age > median	1.077	0.685–1.693	0.748
CAD	1.806	0.778–4.191	0.169
Obesity	1.839	1.116–3.031	<0.05
Hypercholesterinemia	1.014	0.368–2.793	0.978
Smoking	0.862	0.523–1.422	0.562
Hypertension	2.378	0.997–5.670	0.051
Diabetes	1.704	0.950–3.057	0.074
eGFR <60	1.113	0.672–1.842	0.678
Men (*n* = 409)			
EAT > median	2.023	1.338–3.059	<0.001
Age > median	1.063	0.696–1.624	0.776
CAD	2.332	1.394–3.903	<0.05
Obesity	1.397	0.871–2.243	0.166
Hypercholesterinemia	1.100	0.572–2.115	0.775
Smoking	0.744	0.488–1.132	0.167
Hypertension	3.297	1.621–6.705	<0.001
Diabetes	1.020	0.610–1.706	0.940
eGFR <60	1.009	0.639–1.595	0.968

In binary logistic regression, EAT is independently associated with left ventricular hypertrophy. This effect is pronounced in men but absent in women. Median EAT thickness was 4.1 mm (4.1 mm in women, 4.2 mm in men), and median age was 78 years (77.6 years in women, 78.3 years in men). CI, confidence interval of OR; EAT, epicardial adipose tissue; CAD, coronary artery disease; eGFR, estimated glomerular filtration rate.

Aortic valve stenosis and mitral valve insufficiency are established risk factors for LVH (40, 41). Echocardiography was performed using two protocols: a standard program for all participants and an extended program with detailed valve assessment in a subset of 327 participants (19). To assess potential confounding, high-grade valvular disease was included in multivariable linear and logistic regression models as a sensitivity analysis (Supplementary Tables S3, S4). The association between EAT and LVMi remained significant, whereas the association with LVH was significant only in men, likely reflecting the reduced sample size.

## Discussion

In 988 participants aged 70 years and older, increased EAT thickness is associated with advancing age. EAT thickness was dependent on age and metabolic markers, such as BMI and LDL-cholesterol, in older and very old individuals. Neither hypertension nor chronic kidney disease was associated with EAT thickness. As the paracrine activity of EAT has been shown to promote LVH in animal models (9) and EAT has been associated with LVH in younger, healthy populations (13, 15), we investigated whether this association persists in an older population in whom LVH and the resulting diastolic dysfunction represent common clinical challenges. Although EAT thickness was similar between women and men, an association between EAT and both LVMi and LVH was observed only in men.

### EAT thickness in young people

Although methodologies for quantifying EAT thickness differ—most often relying on CT imaging in smaller cohorts—the reported median values span a considerable range. In populations under 74 years of age, thickness measurements have been as low as 0.9 mm (42). In contrast, larger-scale investigations have documented median EAT depths of 4.0 mm and even 5.4 mm, respectively (43, 44). Cohorts with particularly high prevalence of metabolic syndrome (up to 50%) consistently exhibit correspondingly greater EAT accumulation (43). In our study cohort, EAT thickness was independent of sex but increased with advancing age, yielding median values slightly higher than those reported in earlier studies involving younger participants.

### EAT as a component of metabolic disease

EAT is metabolically active and capable of releasing proinflammatory cytokines, adipokines, and other bioactive molecules, which can influence both local myocardial tissue and systemic metabolic processes (5). The observed association between EAT and BMI and LDL-cholesterol in our study aligns with prior research in younger population-based samples, showing an association between EAT and high levels of LDL-cholesterol and high BMI (33, 44–48). The Jackson Heart Study demonstrated a strong association between EAT and metabolic syndrome (*n* = 1,414, OR = 1.48), and Zimmermann et al. showed an association between increased EAT and BMI as well as increased EAT and hyperlipidemia [*n* = 1,217, OR (BMI) = 1.19, OR (hyperlipidemia) = 2.84] (49, 50). Overall, previous and our studies suggest that EAT accumulation reflects broader patterns of metabolic dysfunction.

The association between statin use and EAT thickness observed in this study contrasts with findings from previous reports (35). This effect was predominantly observed in women and may be influenced by the strong association between LDL-cholesterol and EAT in our cohort, as well as the relatively low prevalence of statin use among women (28.9%) despite a median LDL-cholesterol of 151.9 mg/dl.

### Sex differences in the association between EAT and LVH

Given its close anatomical proximity to the myocardium, EAT is positioned to affect cardiac structure and function through paracrine signaling (6, 8). Previous experimental studies have shown that EAT promotes LVH by releasing specific signaling molecules, such as inflammatory cytokines and free fatty acids (4, 5, 9). Epidemiological studies have likewise reported associations between EAT and LVMi, reinforcing the hypothesis that EAT contributes to cardiac remodeling through metabolic and inflammatory pathways (10–18). Our findings extend these experimental and epidemiologic observations by demonstrating a significant association between EAT thickness and LVMi in a population-based sample of older and very old individuals. This suggests that EAT activity may remain relevant in advanced age and continue to contribute to cardiac hypertrophy even in later life.

Interestingly, the association between EAT and LVMi, as well as LVH, was observed only in men and not in women. Other sex-specific differences in EAT have already been shown, such as a stronger association with body fat in women than in men; however, to our knowledge, a difference between men and women regarding the association between EAT and LVH has not been reported yet (44). A possible explanation might be the higher levels of testosterone in men, which are associated with an increased risk of developing left ventricular hypertrophy (51). Molecular evidence also supports sex-dependent differences in myocardial response to pressure overload. In human hearts subjected to chronic pressure overload, transcriptomic profiling and confirmatory analyses have demonstrated that maladaptive left ventricular remodeling is more common in men. In this setting, profibrotic and proinflammatory pathways are upregulated in male myocardium, whereas genes related to extracellular matrix and inflammatory are relatively suppressed in female myocardium. These differences are accompanied by greater histological fibrosis in men than in women, indicating distinct sex-specific regulation of fibrosis and inflammation in left ventricular remodeling (52). This mechanism may also underlie a differential effect of EAT on left ventricular hypertrophy in men; however, direct data supporting this theory are currently lacking, and further research is required.

This sex-specific pattern raises important questions about the underlying mechanisms driving these differences. It is tempting to speculate that the renin–angiotensin system (RAS) may play a role in this discrepancy. In the population-based CARTaGENE study of 19.996 Canadian residents (Québec), 1,284 underwent cardiac MRI as part of the CAHHM study (mean age 55 years). The analyses revealed that renin (but not aldosterone) was associated with cardiac adiposity in women, whereas aldosterone (but not renin) was associated with cardiac adiposity in men (53). The underlying mechanisms are barely understood.

Further research is needed to clarify the contribution of the RAS or other sex-specific factors to the relationship between EAT and LVH.

### Translational implications

Our findings suggest a potential role for EAT as a modifiable target in the prevention and treatment of LVH, particularly in men. Recent cardiometabolic medications, such as glucagon-like peptide 1 receptor agonists and sodium–glucose cotransporter 2 (SGLT2) inhibitors, have been shown to modulate adipose tissue metabolism and improve cardiovascular outcomes.

Given the established links between EAT, metabolic dysfunction, and cardiac remodeling, interventions aimed at reducing EAT thickness or modifying its metabolic activity may offer therapeutic benefits in preventing or reversing LVH. The observed sex-specific differences further suggest that targeted therapies may need to account for these biological differences to optimize outcomes. This consideration is particularly important in the analysis of preclinical animal studies, which often rely exclusively on male or female animals.

## Limitations

Our study has several limitations. Due to the recruitment strategy of our study, there is a bias toward healthier individuals, as participants needed to visit our outpatient clinic. This led to a disproportionately healthy study population, both physically and mentally. Notably, individuals with severely reduced EF (≤30%) were underrepresented in the study population (*n* = 4). Therefore, our findings may not be generalizable to patients with severely impaired systolic function. Severe valve diseases were also underrepresented (*n* = 9/327).

Furthermore, the diagnosis of diabetes in this study was based on medication intake. In case of providers ordering metformin for patients with prediabetes, this study might overestimate the proportion of patients with diabetes in the elderly population.

EAT thickness was measured using echocardiography. This evaluation is time and cost-effective and is not associated with relevant risks but is more dependent on the experience of examiners than MRI or CT scan. All echocardiographic examinations were performed by one of two experienced echocardiographers to minimize interobserver variability, and each measurement was repeated three times in normal heart rhythm and 10 times in arrhythmia to reduce random errors.

Another limitation is the absence of endocrine measurements, which could have provided insights into the hormonal and metabolic pathways underlying the sex-specific differences in the association between EAT and LVH. Future studies should aim to explore the contributions of endocrine factors, including the RAS and sex hormones, to EAT-mediated cardiac remodeling.

## Conclusion

In summary, our study confirms and extends previous findings by demonstrating that EAT thickness increases with age and is influenced by metabolic factors such as BMI and LDL cholesterol. The observed association between EAT and LVH in older adults supports the hypothesis that EAT is involved in cardiac remodeling. The finding that this association is restricted to men suggests the potential presence of sex-specific mechanisms, which warrants further investigation.

## Data Availability

The raw data supporting the conclusions of this article will be made available by the authors, without undue reservation.
