# Supplementary material for: Sex-specific association of epicardial adipose tissue thickness and left ventricular hypertrophy in the older adults—cross-sectional results from the population-based AugUR study
Source: Front Cardiovasc Med. 2026 Feb 18;13:1705319. doi: 10.3389/fcvm.2026.1705319 (PMC12957171; doi:10.3389/fcvm.2026.1705319)
Supplement: Supplementary file 1 [file Datasheet1.pdf]

Supplement

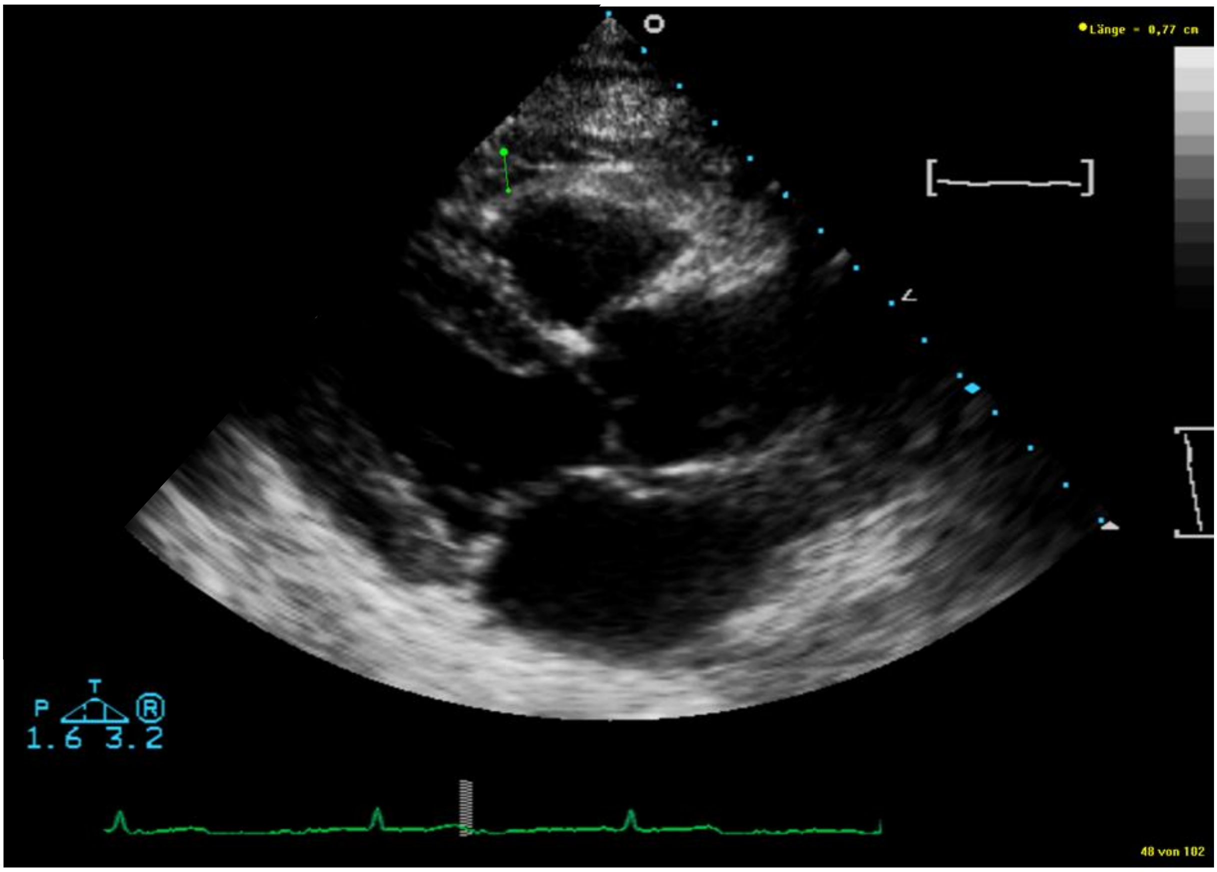

Figure S1: Epicardial adipose tissue measured in parasternal long axis

Epicardial adipose tissue was measured in parasternal long axis at the level of the aortic valve (green dots connected with a line).

	ß	95%CI	p
Age [years]	0.086 mm/year	0.009-0.056	0.007
Sex [0/1 f/m]	0.024 mm	-0.149-0.330	0.457
BMI [kg/m^2]	0.102 mm/kg/m^2	0.076-0.128	< 0.001
LDL-cholesterol [mg/dl]	0.004 mm/mg/dl	0.000-0.007	0.045
Hypertension [0/1 n/y]	0.126 mm/mmHg	-0.143-0.395	0.357
eGFR < 60 [0/1 n/y]	0.388 mm	0.131-0.644	0.003
EF [%]	-0.023 mm/%	-0.022-0.010	0.476
CAD [0/1 n/y]	0.148 mm	-0.168-0.465	0.358
hsCRP [0/1 n/y]	-0.097 mm/mg/dl	-0.173-0.157	0.923
High-grade AVS [0/1 n/y]	0.188mm	-2.185-2.561	0.876

Table S1: Factors associated with epicardial adipose tissue

Univariable analyses of cardio vascular risk factors influencing epicardial adipose tissue

BMI body-mass-index; LDL low-density-lipoprotein; eGFR estimated glomerular filtration rate; EF left ventricular ejection fraction; CAD coronary artery disease; hsCRP high-sensitive C-reactive protein; AVS aortic valve stenosis; 95%CI: 95% confidence interval of ß.

Age groups	70-74	75-79	80-84	85-90	90-95	All (70-95)
**Women n**	154	164	83	26	10	437
**Mean ± SD**	4.09±1.96	3.75±1.81	4.58±1.99	4.64±2.25	4.46±2.71	4.10±1.96
**Minimum**	1.33	1.13	0.90	1.00	1.40	0.90
**5th percentile**	1.60	1.60	1.70	1.00	1.40	1.60
**10th percentile**	1.75	1.82	2.02	1.14	1.49	1.80
**25th percentile**	2.53	2.30	3.07	2.90	2.50	2.47
**Median**	3.77	3.38	4.57	5.22	3.22	3.80
**75th percentile**	5.40	4.78	5.87	6.37	5.80	5.33
**90th percentile**	6.60	6.17	7.14	7.23	9.67	6.57
**95th percentile**	8.13	6.60	7.77	7.37	9.90	7.77
**Maximum**	10.20	9.90	9.80	9.13	9.90	10.20
**Men n**	164	207	112	55	13	551
**Mean ± SD**	4.11±1.85	4.04±1.75	4.53±2.16	4.19±1.76	5.05±2.30	4.20±1.89
**Minimum**	1.20	1.10	1.37	1.30	1.67	1.10
**5th percentile**	1.77	1.90	1.77	1.87	1.67	1.77
**10th percentile**	2.00	2.09	2.21	2.05	1.81	2.07
**25th percentile**	2.62	2.77	2.80	3.03	2.77	2.77
**Median**	3.85	3.63	4.03	3.83	5.57	3.83
**75th percentile**	5.27	5.00	6.13	5.33	6.90	5.40
**90th percentile**	6.53	6.60	7.37	6.79	7.90	6.79
**95th percentile**	7.43	7.17	8.43	7.87	8.17	7.60
**Maximum**	11.37	10.73	12.67	8.53	8.17	12.67

Table S2: EAT [mm] by age groups and sex in 988 participants of the AugUR study

EAT is shown in mm in different age groups and separated for male and female subjects. EAT thickness increases with higher age.

EAT epicardial adipose tissue; AugUR Altersbezogene Untersuchungen zur Gesundheit der University of Regensburg;

		ß	95%CI	p-value
**Total study population (n=327)**	EAT	2.202	0.172-4.232	**0.034**
	Sex	18.205	10.994-25.416	**<0.001**
	Age	0.393	-0.310-1.095	0.272
	CAD	14.277	4.861-23.693	**0.003**
	BMI	1.536	0.688-2.385	**<0.001**
	LDL	0.032	-0.081-0.145	0.576
	Smoking	-1.740	-8.678-5.198	0.622
	Hypertension	12.480	1.665-23.295	**0.024**
	Diabetes	0.049	-8.459-8.557	0.991
	eGFR < 60	0.685	-7.017-8.387	0.861
	High-grade valve disease	5.744	-13.157-24.645	0.550
**Women (n=138)**	EAT	1.639	-1.355-4.632	0.280
	Age	1.055	0.028-2.082	**0.044**
	CAD	15.449	-0.156-31.053	0.052
	BMI	1.257	-0.093-2.421	**0.035**
	LDL	0.014	-0.139-0.167	0.858
	Smoking	-7.232	-17.605-3.141	0.170
	Hypertension	4.672	-12.575-21.919	0.592
	Diabetes	1.005	-11.005-13.014	0.869
	eGFR < 60	2.741	-8.705-14.187	0.636
	High-grade valve disease	11.926	-10.845-34.696	0.301
**Men (n=189)**	EAT	3.704	0.815-6.593	**0.012**
	Age	-0.156	-1.138-0.825	0.753
	CAD	17.762	5.223-30.301	**0.006**
	BMI	1.809	0.532-3.085	**0.006**
	LDL	0.067	-0.103-0.238	0.437
	Smoking	1.946	-7.679-11.571	0.690
	Hypertension	17.572	3.155-31.990	**0.017**
	Diabetes	-2.893	-15.214-9.428	0.643
	eGFR < 60	-1.156	-11.744-9.433	0.829
	High-grade valve disease	-16.137	-49.173-16.900	0.336

Table S3 In multivariable linear regression EAT is associated with left ventricular mass index

In linear regression, EAT is independently associated with left ventricular mass. This effect is more pronounced in male than in female subjects. *CI confidence interval of ß; EAT epicardial adipose tissue; BMI body-mass-index; LDL low density lipoprotein; CAD coronary artery disease; eGFR estimated glomerular filtration rate; High-grade valve disease presence of a high-grade aortic valve stenosis of a high-grade mitral valve insufficiency;*

		**OR**	**95%CI**	**p-value**
**Total study population (n=327)**	EAT > median	1.570	0.911-2.705	0.104
	Sex	0.894	0.502-1.591	0.704
	Age > median	1.118	0.647-1.934	0.690
	CAD	2.834	1.329-6.046	**0.007**
	Obesity	2.010	1.093-3.696	**0.025**
	Hypercholesterinaemia	1.267	0.488-3.292	0.627
	Smoking	0.708	0.398-1.258	0.239
	Hypertension	3.931	1.606-9.622	**0.003**
	Diabetes	1.063	0.538-2.099	0.860
	eGFR < 60	1.294	0.701-2.389	0.409
	High-grade valve disease	1.180	0.261-5.339	0.830
**Women (n=138)**	EAT > median	0.960	0.421-2.189	0.922
	Age > median	1.147	0.497-2.648	0.748
	CAD	3.232	0.786-13.298	0.104
	Obesity	3.016	1.111-8.191	**0.030**
	Hypercholesterinaemia	1.181	0.151-9.252	0.874
	Smoking	0.612	0.235-1.592	0.314
	Hypertension	1.900	0.434-8.324	0.394
	Diabetes	1.403	0.519-3.792	0.504
	eGFR < 60	1.131	0.418-3.065	0.808
	High-grade valve disease	2.972	0.426-20.719	0.272
**Men (n=189)**	EAT > median	2.557	1.184-5.522	**0.017**
	Age > median	1.184	0.549-2.557	0.666
	CAD	3.579	1.334-9.599	**0.011**
	Obesity	1.909	0.827-4.409	0.130
	Hypercholesterinaemia	1.460	0.463-4.606	0.518
	Smoking	0.732	0.337-1.588	0.429

	Hypertension	7.371	2.146-25.324	**0.002**
	Diabetes	0.674	0.245-1.851	0.444
	eGFR < 60	1.246	0.547-2.841	0.601
	High-grade valve disease	0.195	0.015-2.511	0.210

Table S4 In multivariable binary logistic regression EAT is associated with left ventricular hypertrophy

In binary logistic regression, EAT is independently associated with left ventricular hypertrophy. This effect is pronounced in men, but absent in women. Median EAT thickness was 4.1mm (4.1mm in women, 4.2mm in men), median age was 78 years (77.6 years in women, 78.3 years in men)

CI confidence interval of OR; EAT epicardial adipose tissue; CAD coronary artery disease; eGFR estimated glomerular filtration rate; High-grade valve disease presence of a high-grade aortic valve stenosis of a high-grade mitral valve insufficiency;
