# Supplementary material for: Sex-specific association of epicardial adipose tissue thickness and left ventricular hypertrophy in the older adults—cross-sectional results from the population-based AugUR study
Source: Front Cardiovasc Med. 2026 Feb 18;13:1705319. doi: 10.3389/fcvm.2026.1705319 (PMC12957171; doi:10.3389/fcvm.2026.1705319)
Supplement: Supplementary file 2 [file Presentation1.pptx]

## Slide 1
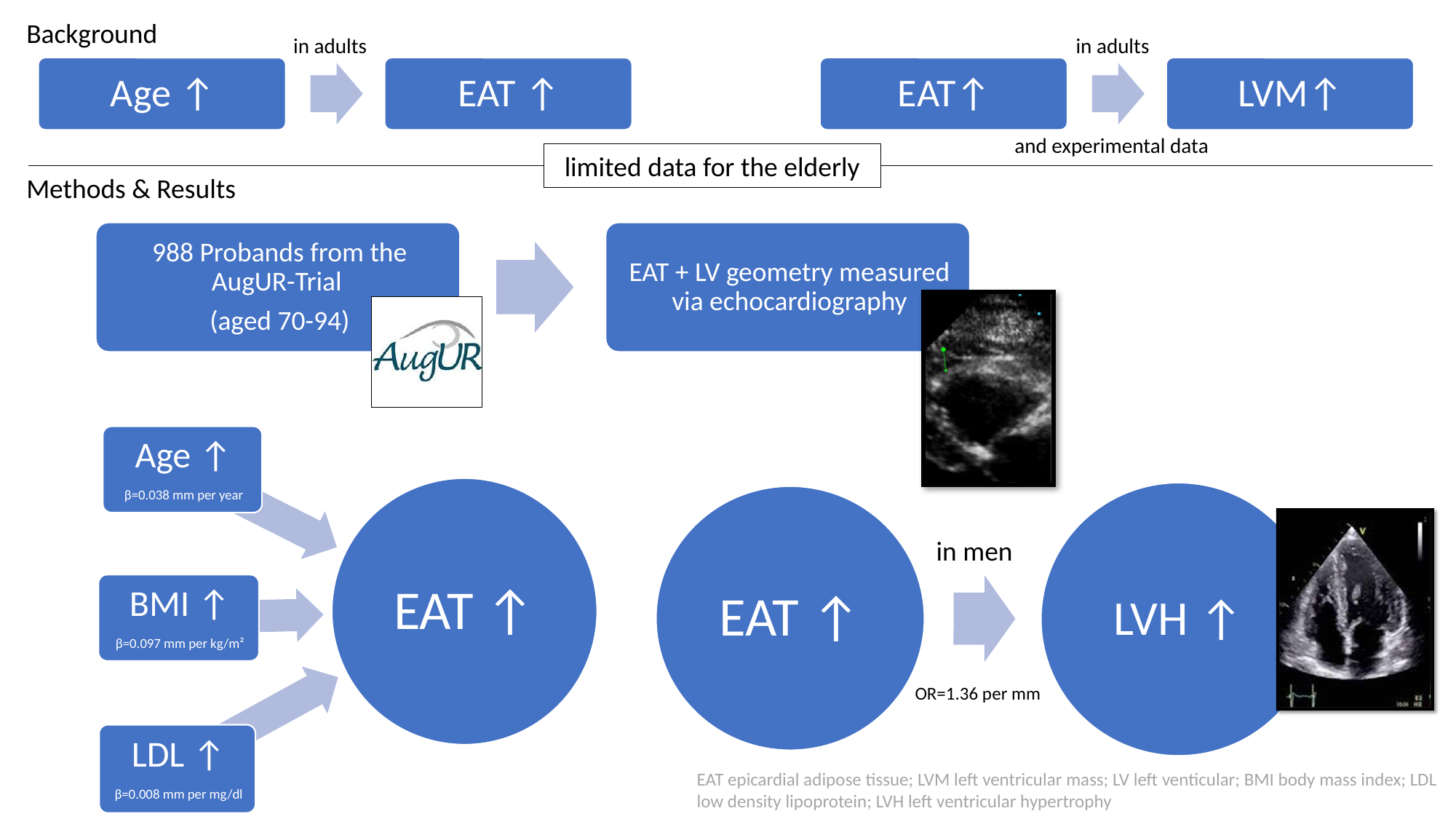

Background
in adults
in adults
and experimental data
limited data for the elderly
Methods & Results
in men
 OR=1.36 per mm
EAT epicardial adipose tissue; LVM left ventricular mass; LV left venticular; BMI body mass index; LDL low density lipoprotein; LVH left ventricular hypertrophy
